# Ewing's Sarcoma of the Zygomatic Arch Presenting in a 69-Year Old: An Unusual Case Report

**DOI:** 10.1155/2011/484976

**Published:** 2011-11-24

**Authors:** Joanna C. Mennie, Robin Reid, Fiona Cowie, Omar Hilmi

**Affiliations:** ^1^Department of Plastic Surgery, St John's Hospital at Howden, West Lothian, Livingston EH54 6PP, UK; ^2^Department of Pathology, Western Infirmary, Glasgow G11 6NT, UK; ^3^Department of Oncology, Beatson West of Scotland Cancer Centre, Glasgow G12 0YN, UK; ^4^Department of Otolaryngology, Head and Neck Surgery, Glasgow Royal Infirmary, Glasgow G4 0SF, UK

## Abstract

*Objective*. We report a rare case of Ewing's sarcoma of the zygomatic arch presenting in a 69-year-old patient. *Method*. Case report and a review of the world literature on Ewing's sarcoma incidence and management. *Results*. Ewing's sarcoma is a malignant round cell tumour of neuroectodermal origin that typically presents in the pelvis and long bones of children and adolescent boys. This report is the first to document the presentation of ewing's sarcoma of the zygomatic arch in a 69-year-old lady. Our patient underwent surgical excision and radiotherapy and at 4-year followup has no signs of recurrence or metastasis. *Conclusion*. To our knowledge this is the first case report to document Ewing's sarcoma of this location in a 69-year-old patient. This case report highlights the importance of diagnostic investigations in Ewing's sarcoma and discusses the management issues that this rare presentation raises.

## 1. Introduction

This is the first report to document Ewing's sarcoma presenting in the zygomatic arch in a patient of 69 years.

Ewing's sarcoma of the bone is an undifferentiated round cell tumour of neuroectodermal origin. It is classified along with extraskeletal Ewing's sarcoma, Askin's tumour, and peripheral neuroectodermal tumour (PNET) into Ewing's family of tumours, first described by Ewing in 1921 [1]. 

Ewing's sarcoma most commonly presents in children; incidence after the 3rd decade accounts for only 10% of cases and is limited to only a few case reports in those patients older than 60. Males have been shown to have a slight preponderance over females in developing the tumour, and the pelvis and long bones are the most common osseous primary site. Only 3% of cases involve the bones of the head and neck [2]. 

## 2. Case Report

In April 2007, a 69-year-old lady presented to the neck lump clinic with a right sided preauricular swelling. The swelling had been present for 26 weeks gradually increasing in size. Examination revealed a firm immobile mass located near the lateral border of the orbit in the superior masseter region. There was no cervical lymphadenopathy, and examination of the oral cavity, larynx, and pharynx was normal. There was no facial nerve weakness. The initial proposed diagnosis was of a metastatic tumor.

**Figure 1:**
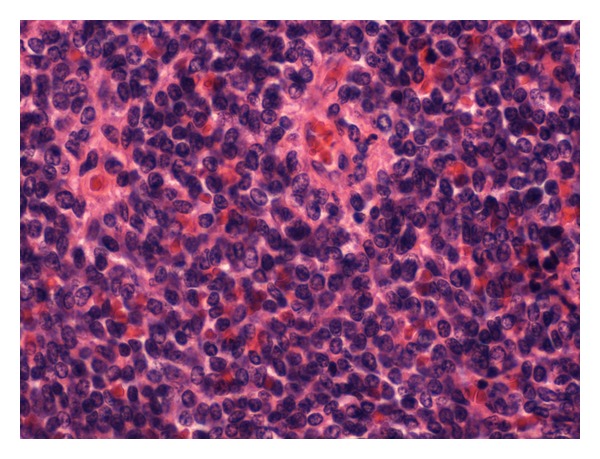
High power H and E image of a monotonous small round blue cell infiltrate.

**Figure 2:**
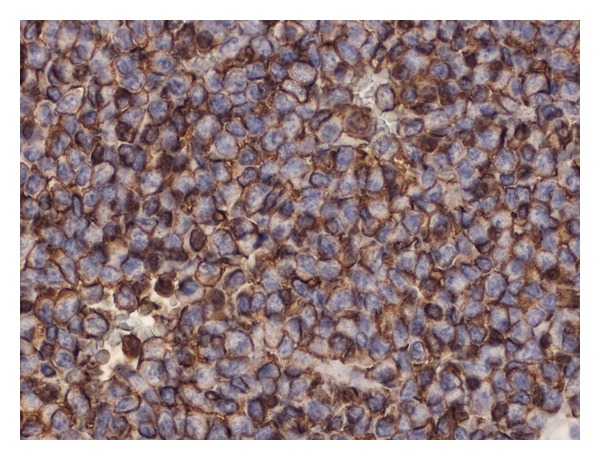
Crisp membrane staining with CD99.

Ultrasound showed the swelling to be 3 cm in diameter and homogeneous in nature. A guided FNA reported suspicious cytology showing sheets of undifferentiated cells amongst an infiltrate of uniform small blue cells with a high nuclear-cytoplasmic ratio (Figure 1). Given this result and considering the patient's age and anatomical site the provisional diagnosis was lymphoma. A guided core biopsy was performed to confirm the diagnosis histologically. The morphological features, however, with supporting immunohistochemical expression of the CD99/MIC2 antigen led to a diagnosis of Ewing's sarcoma (Figure 2). 

**Figure 3:**
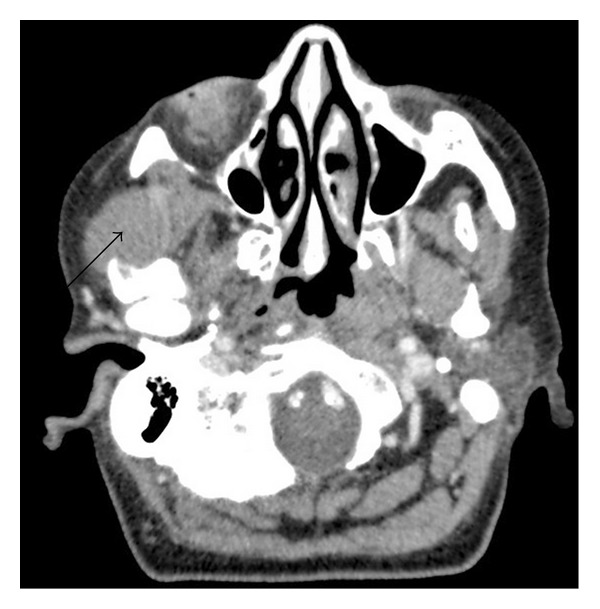
CT axial section showing tumour invading right zygomatic arch.

A single-photon emission computed tomography (SPECT) scan demonstrated an increased tracer uptake in the right zygomatic arch with the bone being atrophic and irregular, eroded by a 2.9 × 1.6 cm nodule adjacent to the temporalis muscle (Figure 3). A staging CT scan of the neck, chest abdomen, and pelvis confirmed no metastasis. 

Following multidisciplinary team (MDT) discussion the decision was made to recommend surgery, with preservation of the facial nerve, to reduce tumour load followed by local radiotherapy. It was felt that neoadjuvant chemo/radiotherapy to shrink the tumour would not be appropriate due to the patients comorbidity (the patient was ASA grade IV).

Using a nerve monitor/stimulator throughout, a pre-auricular incision was made and extended up into the hairline with anterior extension along a skin crease. A skin flap was elevated anteriorly and the temporal branch of the facial nerve identified overlying the zygomatic arch. The zygomatic branch was then defined by retrograde dissection. The tumour was found to be pushing up on the underlying fat pad and involving the masseter muscle and zygoma. After anterior mobilization of the facial nerve the involved portion of the zygoma and associated tumour was removed. All branches of the facial nerve were identified and tested prior to closure; all were intact.

Histopathology confirmed the diagnosis of Ewing's sarcoma. Significant amounts of intracellular glycogen were present, and the sample was strongly positive for CD99/MIC2.

Molecular genetic analysis performed by fluorescence in situ hybridization (FISH) revealed the translocation t(11:22) that is found in >90% of Ewing's sarcoma.

Postoperatively the patient recovered well other than a slight neuropraxia of the temporal branch of the facial nerve.

Two months later the patient underwent 54 Gy of external beam radiotherapy in 27 fractions. The only complication in our patient was a right ear infection and perforation followed by a mild right-sided sensorineural hearing deficit. She was treated with Gentisone HC and fitted with a hearing aid.

Subsequently the patient has been followed up for 4 years. Facial nerve function has returned to normal and there are no significant cosmetic issues. CT and MRI coregistration scans show no evidence of local recurrence or metastatic spread.

## 3. Discussion

Primary Ewing's sarcoma involving the head and neck accounts for only 3% of Ewing's sarcoma cases of which the most commonly affected areas are the skull, maxilla, and mandible. The zygomatic region has only been reported in 6 previous cases to our knowledge in patients aged 2–31 years old [3] with a mean age of 11 years. Incidence of Ewing's sarcoma over the age of 60 is reported to be <2% in one 18 yr study and here we present the oldest case of head and neck Ewing's sarcoma [4]. This case is therefore unique when considering not only the involvement of the zygomatic arch but also the patient's age and thus poses a diagnostic difficulty to the consulting physician.

Lymphoma would be a commoner cause of such a presentation in this age group, with cytological features that can easily be confused with Ewing's sarcoma. This case demonstrates the advantage of guided core biopsy in obtaining histopathological material over the more traditional open biopsy that in this case could have had significant management implications including the need for a wider surgical excision. This case also highlights the importance of a broad differential diagnosis and utilization of investigations such as immunohistochemistry and molecular genetics to facilitate a prompt and accurate diagnosis. Misdiagnosis like one previously reported case can be fatal due to the aggressive nature of the tumour and the poor prognosis associated with metastatic disease [2, 5]. 

Accurate diagnosis is synergistic with appropriate treatment to improve overall survival. Studies have shown that Ewing's sarcoma requires risk-adapted treatment taking into account the tumour location, size, and presence or absence of metastasis. Those that are considered high risk should undergo high-dose intensive chemotherapy and radiotherapy if fit. Recent research has shown the combination of surgery, radiotherapy, and chemotherapy has been proven to yield best results, with 5-year survival reported as reaching 55–75%, a great improvement from the 10% which was achieved with single modality therapy in the 1970s [6]. However, surgical excision and radiotherapy pose a problem when, like our patient, the head and neck is involved.

Firstly surgery may result in gross deformity and compromise facial function in order to obtain clear margins, especially if tumours are large or located near intricate structures. Each case needs to be considered individually in terms of tumour location and size along with the psychological effect that major facial surgery would have on the patient. It is well documented the negative impact facial disfigurement can have on a person's life especially in adolescents as well as the discrimination they face from the public, therefore such surgical excision requires a multidisciplinary approach with the option for reconstructive surgery explored early. Our patient had a small tumour in comparison to others noted and surgical excision without reconstruction was appropriate.

The other issue this case raises about head and neck Ewing's sarcoma is the suitability of radiotherapy as part of treatment. Radiotherapy to the head and neck can result in many undesirable side effects. In younger patients the brain and facial skeleton is rapidly growing and exposure to radiotherapy may result in significant long-term side effects. Patients of any age may also experience adverse effects such as sensorineural hearing loss, cataracts, temporomandibular joint ankylosis, and secondary tumours. The standard treatment approach for Ewing's sarcoma is initial chemotherapy, followed by surgery if possible then further chemotherapy. Radiotherapy is added if surgery is not possible or if the surgery or chemotherapy results are not adequate. Disease-free survival has occasionally been reported after surgical excision alone and one such study has suggested that radiotherapy should be restricted to cases where margins at surgery are not clear or in cases of recurrence [7, 8]. However, as documented by Ewing, Ewing's sarcoma is extremely radiosensitive and as mentioned local radiotherapy has been reported to allow a better overall survival [1, 6]. In addition, radiotherapy may be the only option in older patients who are likely to present with comorbidities and may not show the resilience that younger patients do to chemotherapy. In our patient comorbidities ruled out chemotherapy so radiotherapy was used alone, unfortunately; however, sensorineural hearing loss did occur as a side effect. 

## 4. Conclusion

Lumps in the head and neck are a common presentation at ENT clinics. This case highlights the importance of maintaining a wide differential diagnosis in these cases and the importance of utilizing the appropriate investigations to obtain an accurate diagnosis. US-guided core biopsy together with immunohistochemistry reduces misdiagnosis and helps avoid the unnecessary complications that ensue, especially when the presentation is rare.
